# Knowledge of patients’ visual experience during cataract surgery: a survey of eye doctors in Karachi, Pakistan

**DOI:** 10.1186/1471-2415-12-55

**Published:** 2012-11-08

**Authors:** Mohammad Zain Tauqir, Tanveer Anjum Chaudhry, Sehreen Mumtaz, Khabir Ahmad

**Affiliations:** 1 Section of Ophthalmology, Department of Surgery, Aga Khan University, Stadium Road, P.O. Box 3500, Karachi, 74800, Pakistan

## Abstract

**Background:**

Several recent studies have recommended that ophthalmologists must be aware of the visual sensations (and their associated anxiety/fear) experienced by patients undergoing cataract surgery. We assessed the knowledge of a group of eye doctors in Pakistan regarding these phenomena.

**Methods:**

This was a cross-sectional survey. Eye doctors (ophthalmologists, residents and medical officers) attending the Ophthalmological Society of Pakistan Annual Conference 2011, in Karachi were invited to participate in the study. A self-administered structured questionnaire was used to examine their knowledge of visual sensations and their associated anxiety/fear experienced by patients during cataract surgery. Simple frequencies and proportions were calculated to describe the data.

**Results:**

A total of 150 ophthalmologists, residents and medical officers were invited to participate in the study. Of these, 68 (45.3%) responded. The mean age (±SD) of the participants was 42.9 (13.2) years. The proportion of participants who thought that patients could experience visual sensations during cataract surgery under regional anaesthesia was 89.7% and that under topical anaesthesia was 73.5%. The most frequently cited sensations included: light perception, changes in light brightness, movements, instruments and surgeon’s hands or fingers.

The eye doctors estimated that 38.9% and 64.3% patients would see at least something during cataract surgery under regional anaesthesia and topical anaesthesia, respectively. They also believed that 24.2%-36.9% of patients may experience anxiety/fear as a result of visual sensations during such surgery. Approximately half of the eye doctors did not think that retained vision was a source of fear or anxiety for the patients. While most of them acknowledged the importance of preoperative counselling in helping to alleviate such fear/anxiety, the majority of them did not regularly counsel their patients on what to expect during the surgery.

**Conclusion:**

Our study reveals that a significant proportion of eye doctors do not have adequate knowledge of the visual phenomenon and their associated anxiety or fear, that patients can experience during cataract surgery. Targeted educational interventions are needed to increase awareness of this phenomenon among eye care professionals.

## Background

Cataract is the leading cause of treatable blindness and visual impairment worldwide [1] and cataract surgery is one of the most frequently performed surgeries in both developed and developed countries [2]. Several recent studies show that individuals undergoing cataract surgery retain some form of vision during the surgery [3-7]. When patients were asked by different investigators what they saw during the surgery, they reported seeing light, one or more colours, surgical instruments or surgeons’ hands. These experiences can be frightening for some of the individuals undergoing cataract surgery and may in some cases cause some unforeseen complications. It is important that eye surgeons are aware of patients’ visual experiences during surgery. A nationwide study in the UK revealed that more than half (54%) surgeons believe that patients do not experience any perception of light during the surgery [8]. However, different studies reveal that about 80-100% patients retain some form of vision during the surgery [4-6,9,10]. Such a gap of knowledge is striking. To address patients’ fears, the role of preoperative counselling has been researched with encouraging results [11]. To the best of our knowledge, research on these aspects of cataract surgery is lacking in Pakistan and other developing countries, where cataract is the leading cause of blindness and visual impairment and where surgical services have expanded substantially in the last one and a half decade. We assessed the knowledge of ophthalmologists in Pakistan’s Karachi city regarding visual experiences of the patients during cataract surgery under regional and topical anaesthesia.

## Methods

This was a cross-sectional survey conducted at the Ophthalmological Society of Pakistan Annual Conference 2011, in Karachi. Participants were practising eye doctors (ophthalmologists, postgraduate trainees and medical officers) attending the event. They were recruited using convenience sampling method. Using a structured, self-administered questionnaire, participants were asked about their demographics, training, work experience and their knowledge of visual perceptions experienced by patients during cataract surgery under regional and topical anaesthesia. The questions regarding visual perceptions were adopted from published studies [8-14] to facilitate comparison and extension of the findings. The salient questions were these: Do you think patients could experience visual sensations during cataract surgery and, if yes, which of the following sensations might be experienced? 1) flashes of light 2) change in brightness of the light 3) colours 4) movements 5) instruments 6) surgeon’s hands or fingers. What percentage of patients do you believe would experience visual sensations during cataract surgery? What percentage of patients do you believe would experience anxiety/fear by what they see? Do patients tell you that they were frightened by what they saw during surgery? Do you counsel patients about what visual experience to expect? Do you think there is a need for such a counselling?

Approval for the survey was taken from the Ethics Review Committee at Aga Khan University, Karachi. An informed written consent was obtained from the participants. Data were analysed using IBM SPSS Statistics 19. Simple frequencies and proportions were calculated to describe the data.

## Results

A total of 150 eye doctors were contacted during the two-day event. Of these, 68 (45.3%) responded. Majority of the participants were male ophthalmologists of Mohajir and Sindhi ethnic origin. Most of them were based in Karachi and Hyderabad (Table 1). The mean age (±SD) of the participants was 42.9 (13.2) years. The proportion of participants who thought that patients could experience visual sensations during cataract surgery under regional anaesthesia was 89.7% and that under topical anaesthesia was 73.5%. The most frequently cited sensations were light perception, changes in light brightness, movements, instruments and surgeon’s hands or fingers (Table 2).

**Table 1:** Selected characteristics of participant eye doctors (n= 68)

**Variable**	**No.**	**%**
**Age (years)**		
≤ 29	10	14.7
30-39	20	29.4
40-49	22	32.4
≥ 50	16	23.5
**Gender**		
Male	52	76.5
Female	16	23.5
Ethnicity*		
Mohajir	41	60.3
Sindhi	14	20.6
Others	5	7.3
**City of practice**		
Karachi	57	83.8
Hyderabad	7	10.3
Others	4	5.9
**Position**		
Consultant	46	67.6
Resident year 1-2	7	10.3
Resident year 3-4	13	19.1
Medical Officer	2	2.9
**Work practice type**		
Government service only	19	27.9
Private practice only	17	25.0
Charitable hospital only	8	11.8
Private practice and government hospital	10	14.7
Private practice and charitable hospital	13	19.1
All three	1	1.5
**Perform cataract surgery**		
Yes	64	94.1
No	4	5.9

*8 participants (11.8%) refused to provide information about their ethnicity.

**Table 2:** Eye doctors’ responses to the question: Do you think patients could experience visual sensations during cataract surgery, and if yes, which sensations might be experienced?

Question*	**Response**	**Freq.**	Percent**
Do you think patients could experience visual sensations during cataract surgery under regional anaesthesia? If yes, which of the following sensations might be experienced?	**Yes**	**61**	**89.7**
	Light perception	41	67.2
	Flashes of light	10	16.4
	Change in brightness of the light	24	39.3
1. flashes of light	Colors	11	18.0
2. change in brightness of the light	Movements	28	45.9
3. colours	Instruments	12	19.7
4. movements	Surgeon’s hands/fingers	14	23.0
5. instruments	**No**	**7**	**10.3**
6. surgeon’s hands/ fingers			
Do you think patients could experience visual sensations during cataract surgery under topicalanaesthesia? If yes, which of the following sensations might be experienced?	**Yes**	**50**	**98.0**
	Light perception	29	58.0
	Flashes of light	8	16.0
1. flashes of light	Change in brightness of the light	23	46.0
2. change in brightness of the light	Colors	12	24.0
3. colours	Movements	30	60.0
4. movements	Instruments	16	32.0
5. instruments	Surgeon’s hands/fingers	15	30.0
6. surgeon’s hands/ fingers.	**No**	**1**	**2.0**

* A total of 68 eye doctors participated in the study. Some questions were left unanswered by a number of respondents. Cases with missing data were not included in the analyses, resulting in different sample sizes for different parts of analysis.

** Percentages add to more than 100 because participants could mention more than one type of visual perception.

The eye doctors believed that 38.9% and 64.3% patients would see at least something during cataract surgery under regional anaesthesia and topical anaesthesia, respectively. They also believed that 24.2%-36.9% of such patients would experience anxiety/fear as a result of visual sensations (Table 3). While most eye doctors thought that preoperative counselling could reduce patients’ anxiety, less than half reported that they routinely (always or most of the time) counselled their patients for possible visual phenomenon (Table 4).

**Table 3:** Percentage of patients that eye doctors (n=68) believed would experience visual sensations and anxiety/fear during cataract surgery

**Question**	**% ± SD**
What percentage of patients do you believe would experience visual sensations during cataract surgery under regional anaesthesia?	38.9 ± 29.7
What percentage of patients do you believe would experience anxiety/fear by what they see during cataract surgery under regional anaesthesia?	24.2 ± 9.5
What percentage of patients do you believe would experience visual sensations during cataract surgery under topical anaesthesia?	64.3 ± 24.5
What percentage of patients do you believe would experience anxiety/fear by what they see during cataract surgery under topical anaesthesia?	36.9 ± 12.7

**Table 4:** Eye doctors’ responses to questions regarding preoperative counseling of patients about retained vision during cataract surgery as source of fear or anxiety

		**Type of anesthesia**			
		***Regional***		***Topical***	
Question*		**No.**	**%**	**No.**	**%**
Is retained vision during surgery a source of fear or anxiety for patients?	Yes	29	42.6	26	54.2
	No	39	57.4	22	45.8
	***Total***	**68**	**100.0**	**48**	**100.0**
Do patients tell you that they were frightened by what they saw during cataract surgery?	Always	0	0.0	2	4.2
	Most of the time	3	4.6	7	14.6
	Sometimes	15	23.1	17	35.4
	Rarely	30	46.2	12	25.0
	Never	17	26.2	10	20.8
	***Total***	**65**	**100.0**	**48**	**100.0**
Do you counsel patients about what visual experience to expect?	Always	11	17.2	23	46.9
	Most of the time	11	17.2	8	16.3
	Sometimes	16	25.0	4	8.2
	Rarely	11	17.2	5	10.2
	Never	15	23.4	9	18.4
	***Total***	**64**	**100.0**	**49**	**100.0**
Do you think there is a need for such counseling?	Yes	54	81.8	42	87.5
	No	12	18.2	6	12.5
	**Total**	**66**	**100.0**	**48**	**100**

* A total of 68 eye doctors participated in the study. Some questions were left unanswered by a number of respondents. Cases with missing data were not included in the analyses, resulting in different sample sizes for different parts of analysis.

## Discussion

Our study is the first from a developing country to examine the knowledge of doctors regarding the visual sensations and their associated anxiety/fear that patients can experience during cataract surgery. In our study, the surveyed eye doctors reported that, in their opinion, 38.9% and 64.3% patients could see during cataract surgery under regional and topical anaesthesia, respectively. This is a significant underestimation of the proportion of patients who may actually experience visual sensations. Previous studies [3-6,9-14] indicate that 89% to 100% patients operated under topical anaesthesia and 55.7% to 87.5% patients operated under regional anaesthesia retain at least sufficient vision to perceive light.

24.2% to 36.9% patients, the doctors estimated, would experience anxiety/fear due to visual sensations. These percentages are higher than those reported in previous studies [4,5,7,9-11] which showed that such visual sensations are a cause of fear/anxiety in 3% to 19% of the patients.

Patients’ cooperation is essential in all stages of cataract surgery. However, it can be lost due to fear and anxiety, increasing the risk of perioperative complications. Fear and/or anxiety may also result in other adverse events such as increased heart rate and blood pressure, difficulty in breathing, and an acute panic attack. All these events are undesirable considering that many people undergoing cataract surgery are elderly having multiple co-morbidities such as hypertension or ischaemic heart disease [7]. Fear and anxiety due to visual sensations can be prevented by effective pre-operative counselling of patients [11,15]. For example, in a randomized clinical trial [15], Haripriya and colleagues found that patients receiving counselling were significantly less likely to have fears from visual sensations during cataract surgery under topical anaesthesia compared with their counterparts who did not receive such counselling.

However, it was disappointing to note that less than half of the eye doctors in our study regularly counselled their patients preoperatively (32.4% and 45.6% for regional and topical anaesthesia, respectively) which shows that not much attention is being paid to this detail. Although it was encouraging to note that most surgeons had a good knowledge of the different visual sensations patients can experiences during cataract surgery, some participants expressed amazement when they read the questionnaire and even remarked that they had never thought about it (the visual phenomenon).

A limitation of this study was that a significant number of respondents left some questions concerning visual sensations unanswered. Those who did not answer were more likely to be consultants and men. A possible explanation is that the study was conducted during an eye conference when many of the participants were unable to devote sufficient attention and time to complete the survey form. The relatively large number of questions examined could have been overwhelming for some of the participant eye doctors and may have contributed to the missing data.

## Conclusions

Our study indicates that a significant proportion of eye doctors have a poor knowledge of the visual sensations experienced by patient undergoing cataract surgery. We strongly recommend targeted educational interventions to increase awareness of these phenomena and their associated anxiety. Future research would benefit from having a more representative sample, by including eye doctors from other cities in Pakistan in their usual settings, minimizing some of the limitations we faced in our data collection.

## Competing interests

The author(s) declare that they have no competing interests.

## Authors’ contributions

MZT, TAC and KA conceived the report and developed the questionnaire. MZT and SM collected the data and entered it. KA and MZT performed the statistical analysis. MZT and KA drafted the manuscript. TAC and KA and SM contributed to review, and to the revision of the report. All authors read and approved the final manuscript.

## Pre-publication history

The pre-publication history for this paper can be accessed here:

http://www.biomedcentral.com/1471-2415/12/55/prepub
